# Cannabidiol induces autophagy and improves neuronal health associated with SIRT1 mediated longevity

**DOI:** 10.1007/s11357-022-00559-7

**Published:** 2022-04-20

**Authors:** Zhizhen Wang, Peng Zheng, Xi Chen, Yuanyi Xie, Katrina Weston-Green, Nadia Solowij, Yee Lian Chew, Xu-Feng Huang

**Affiliations:** 1 Australian Centre for Cannabinoid Clinical and Research Excellence (ACRE), New Lambton Heights, NSW Australia; 2 Illawarra Health and Medical Research Institute (IHMRI) and School of Medicine, University of Wollongong, Wollongong, NSW 2522 Australia; 3 Molecular Horizons, Faculty of Science, Medicine and Health, University of Wollongong, Wollongong, NSW 2522 Australia; 4 School of Psychology, University of Wollongong, Wollongong, NSW 2522 Australia; 5 Flinders Health and Medical Research Institute and College of Medicine and Public Health, Flinders University, Bedford Park, South Australia 5042 Australia

**Keywords:** Cannabidiol, Aging, Autophagy, Neurite outgrowth, SIRT1/*sir-2.1*, *C. elegans*

## Abstract

Autophagy is a catabolic process to eliminate defective cellular molecules via lysosome-mediated degradation. Dysfunctional autophagy is associated with accelerated aging, whereas stimulation of autophagy could have potent anti-aging effects. We report that cannabidiol (CBD), a natural compound from *Cannabis sativa*, extends lifespan and rescues age-associated physiological declines in *C. elegans*. CBD promoted autophagic flux in nerve-ring neurons visualized by a tandem-tagged LGG-1 reporter during aging in *C. elegans*. Similarly, CBD activated autophagic flux in hippocampal and SH-SY5Y neurons. Furthermore, CBD-mediated lifespan extension was dependent on autophagy genes (*bec-1*, *vps-34*, and *sqst-1*) confirmed by RNAi knockdown experiments. *C. elegans* neurons have previously been shown to accumulate aberrant morphologies, such as beading and blebbing, with increasing age. Interestingly, CBD treatment slowed the development of these features in anterior and posterior touch receptor neurons (TRN) during aging. RNAi knockdown experiments indicated that CBD-mediated age-associated morphological changes in TRNs require *bec-1* and *sqst-1*, not *vps-34*. Further investigation demonstrated that CBD-induced lifespan extension and increased neuronal health require *sir-2.1*/SIRT1. These findings collectively indicate the anti-aging benefits of CBD treatment, in both in vitro and in vivo models, and its potential to improve neuronal health and longevity.

**Supplementary Information:**

The online version contains supplementary material available at 10.1007/s11357-022-00559-7.

## Introduction

Aging is an inevitable biological process that is accompanied by gradual changes in most body systems [1]. The effects of aging are observed in all tissues, including in the nervous system. Current research of the biology of aging and longevity focuses on the cellular and molecular processes involved in the natural process of aging [2], as well as pathological processes that occur in age-related diseases, such as Alzheimer’s disease [3] and Huntington’s disease [4].

One of the cellular processes that are disrupted during aging is autophagy [5]. Autophagy is an evolutionarily conserved process that cells use to deliver cytoplasmic substrates to lysosomes for degradation. During autophagy, double-membraned vesicles called autophagosomes are formed to engulf intracellular components [6]. Autophagosomes fuse with lysosomes to produce autolysosomes, which degrade the autophagic cargo as well as the inner autophagosomal membrane [7]. Defective autophagy has been associated with accelerated aging, likely contributing to the accumulation of damaged macromolecules and organelles. Therefore, the induction of autophagy may have anti-aging effects [8]. Extensive genetic studies have shown that autophagy is linked to multiple processes associated with the regulation of extended lifespan regulation, including caloric restriction [9], AMP-activated protein kinase (AMPK) signaling [10], inhibition of the mechanistic target of rapamycin (mTOR) protein kinase [11], and the insulin/IGF-1 (insulin-like growth factor 1) pathway [12].

Cannabidiol (CBD), a non-intoxicating cannabinoid from *Cannabis sativa*, exhibits a broad spectrum of beneficial pharmacological effects, including antipsychotic [13], pro-cognitive [14], anti-inflammatory [15], and antioxidant [16] properties. Recently, CBD treatment has been shown to increase lifespan and improve healthspan in different models, such as the nematode worm (*Caenorhabditis elegans*) [17, 18] and zebrafish (*Danio rerio*) [19]. However, the exact mechanism through which CBD modulates lifespan is unclear. Furthermore, although CBD has been shown to induce autophagy in cultured neuronal cells [20], it is unknown whether CBD can trigger autophagy, or have another anti-aging effect, in vivo.

*Caenorhabditis elegans* (*C. elegans*) has provided crucial insights into the cellular signaling pathways and molecular mechanisms underlying the process of aging [21]. In *C. elegans*, clear age-dependent morphological changes are widespread across tissue, cellular, and molecular levels [22]. In addition, the short lifespan and features of *C. elegans* nervous system are favorable for studies of neuronal or whole organismal aging [23].

In the present study, we demonstrate that autophagy is required in the regulation of longevity and age-associated neuronal changes during CBD treatment. Furthermore, we show that CBD-mediated mechanisms are involved in autophagy in vitro, using a human neuronal cell line and mouse primary cultured hippocampal neurons. As autophagy plays an important role in several age-related diseases, our findings provide evidence that CBD extends lifespan and modulates neuronal aging through the SIRT1/*sir-2.1*-autophagy pathway.

## Methods

### C. elegans strains, maintenance conditions, and pharmacological treatment

*C. elegans* strains used in this study are listed in Table S1. Strains were provided by the *Caenorhabditis* Genetics Center (CGC), University of Minnesota (Minneapolis, MN, USA). All strains were maintained on nematode growth medium (NGM) agar plates seeded with a lawn of *E. coli* strain OP50 at 20℃ unless otherwise noted. For all the assays, synchronized worms were prepared by alkaline hypochlorite treatment (bleach treatment), as previously described [24].

CBD was purchased from THC Pharm (Frankfurt, Germany) in powder form. CBD was dissolved in 100% DMSO to produce a stock solution at 10 mM concentration (stored at − 20℃) and diluted immediately prior to pouring the plates. Worms were exposed to CBD throughout their lifespan (i.e., from eggs until death) unless otherwise noted.

### Lifespan assay

The lifespan assay was performed at 20 ℃ using standard NGM plates seeded with *E. coli* OP50 or dsRNA-expressing bacteria with 15–20 worms per plate. Worms were counted and transferred to new plates daily. The L4 larval stage was recorded as day 0, while death was determined through an absence of movement with and without stimulation. Worms displaying internal hatching were censored. The lifespan experiment was performed at least twice (details shown in Supplementary Table S2).

### Pharyngeal pumping rate

To quantify pharyngeal pumping rate, 20 synchronized adult worms per group were transferred to NGM agar plates. After 1, 3, and 5 days of adulthood, the pharyngeal pumping rate was counted for 30 s by manual observation, with one pump defined as one contraction of the posterior bulb/grinder. Experiments were performed in duplicate.

### Fertility assay

The fertility assay was performed using 20 L4 synchronized worms per group. A single young adult worm was transferred to a new NGM agar plate with *E. coli* OP50. Each worm was allowed to lay eggs at 20℃ for 24 h. The next day, the adult worm was transferred to a new NGM plate, and the steps were repeated for 5 days. The number of hatched worms was recorded after 2 days. Experiments were performed in duplicate.

### Body bend assay

Age-synchronized worms were placed on unseeded NGM plates and allowed to acclimatize for 5 min. The number of body bends was recorded at room temperature and counted for 30 s. A complete body bend was defined as the bending of the head region across the centerline of the animal, in which a full sinusoid was completed.

### Neuronal aging experiments

Neuron aging assays were performed using the *C. elegans* strain *zdIs5[Pmec-4::GFP]*, which expresses green fluorescent protein (GFP) in the six touch receptor neurons. Age-synchronized worms were cultured on plates at 20 ℃ and transferred to new plates daily until the assay was completed. Worms were considered deceased when pharyngeal pumping ceased and touch response was not apparent. On days 1, 5, 8, and 12 of adulthood, we randomly selected individuals from an asynchronous population to monitor aging neurons. To score the incidence of aberrant neuronal structures in touch neurons, we measured ALM and PLM aberrations, as described by [25, 26]. Worms were paralyzed with 1 M of sodium azide solution and then mounted on an agarose pad. Images were captured using a DMI6500B laser scanning confocal microscope. A minimum of 20 worms per group were measured at each time point. All RNAi feeding was carried out from the egg during the whole life unless otherwise indicated.

### Autophagy measurements in C. elegans

Autophagy was monitored by counting GFP-positive LGG-1 punctae in body-wall muscle, proximal intestinal cells, and terminal pharyngeal bulb of strain **DA2123** *adls2122* [*lgg-1p::GFP::lgg-1* + *rol-6*] and the nerve-ring neurons of strain **MAH242** *sqls24* [*rgef-1p::GFP::lgg-1* + *unc-122p::RFP*]. The puncta were counted using ImageJ Software (ComDet v.0.3.7 plug-in, Rasband, W.S., ImageJ, U.S. National Institutes of Health, Bethesda, Maryland, USA). A minimum of 20 worms per group were measured at each time point.

Autophagic flux was monitored by counting mCherry-GFP-LGG-1 punctae in the terminal pharyngeal bulb of strain **MAH215** *sqls11*[*lgg-1p::mCherry::GFP::lgg-1* + *rol-6*] and the nerve-ring neurons of strain **MAH508** *sqEx67*[*rgef-1p::mCherry::GFP::lgg-1* + *rol-6*]. Images of mCherry-GFP-LGG-1 punctae were scored using the same method as examining GFP::LGG-1 puncta. The number of autophagosomes (APs) was calculated as the GFP-positive puncta, and the number of autolysosomes (ALs) was calculated as the difference between the mCherry-positive puncta and the mCherry-GFP-positive puncta. A minimum of 20 worms per group were measured at each time point.

### RNAi interference experiments in C. elegans

Gene silencing was performed by feeding *C. elegans* with HT115 bacteria transformed with L4440 vector for expression of dsRNA of genes of interest. The following RNAi clones from Ahringer Library [27] were used: L4440 empty vector control, *bec-1* (T19E7.3), *sqst-1* (T12G3.1), *vps-34* (B0025.1), and *sir-2.1* (R11A8.4). RNAi clones were grown overnight at 37℃ in Luria broth (LB) with 50 µg/ml ampicillin at 200 rpm. For RNAi feeding plates, NGM plates were prepared with the addition of 25 µg/ml carbenicillin and 1 mM isopropyl β-D-1-thiogalactopyranoside (IPTG) to induce dsRNA expression. RNAi feeding was conducted throughout the lifespan of the worms unless otherwise indicated.

### Cell culture and treatment

Human neuroblastoma SH-SY5Y (ATCC CRL-2266) cells were maintained at 37℃ in a humidified incubator with 5% CO_2_ and 95% relative humidity. SH-SY5Y cells were cultured in Dulbecco’s modified eagle medium (DMEM)/F12 supplemented with 10% of heat-inactivated fetal bovine serum (FBS) and 1% penicillin–streptomycin from Bovogen Biologicals (Victoria, Australia). SH-SY5Y cells were differentiated, as described previously [28]. Briefly, cells were seeded in MaxGel ECM (E0282, Sigma-Aldrich, Sydney)–coated 96-well plates with media containing 10 µM retinoic acid (RA, Sigma-Aldrich, Sydney) in the dark. Cells were observed for neurite outgrowth using the IncuCyte ZOOM live-cell imaging system (Essen BioScience, UK). Neurite integrity was quantified using the automated NeuroTrack module of the IncuCyte ZOOM system for live-cell analysis. Furthermore, differentiated cells were exposed to various concentrations of CBD (0.1, 0.5, 1, 2.5, 5, 10 µM) in 0.1% DMSO and incubated for 24, 48, and 72 h.

### Primary hippocampal neuron culture

Primary hippocampal neuron cultures were prepared using methods described previously, with slight modifications. Briefly, hippocampal neurons were collected from postnatal (P0–P2) C57BL/6 mice and gently dissociated with a plastic pipette. After trypsinization for 30 min, the supernatant containing hippocampal cells was centrifuged at 100 × *g* for 5 min, and the pelleted cells were collected. The hippocampal cells were plated in a Neurobasal medium (GIBCO, Invitrogen) supplemented with B27 (GIBCO, Invitrogen), 20 mM L-glutamine (Sigma-Aldrich), 100 U/ml penicillin, and 100 µg/ml streptomycin. After 24 h of culture, 10 µM 5-fluoro-2′-deoxyuridine (5-FDU) was added to halt the growth of non-neuronal cells. The cells were plated in 24-well dishes containing glass coverslips coated with poly-D-lysine at a density of 5 × 10^4^ cells per well for immunofluorescence assay. Cells were maintained in a humidified 5% CO_2_ atmosphere at 37 ℃, with half media changes twice weekly. On the seventh day (7 days in vitro (DIV)), neurons were treated with or without 1 µM CBD for 24 h. All experimental procedures were approved by the Animal Ethics Committee, University of Wollongong, Australia (AE19/15).

### Western blot

After various treatments, cells were harvested in lysis buffer containing NP40, Protease Inhibitor Cocktail (Sigma-Aldrich), 1 mM of phenylmethylsulfonyl fluoride (Sigma-Aldrich), and 0.5 mM of β-glycerophosphate (Sigma-Aldrich). Protein concentration was determined using the DC assay (Bio-Rad, Hercules, USA). Proteins (20 µg) were subjected to SDS-PAGE (120 V for 90 min), then transferred to nitrocellulose membranes (GE Health, Chicago, USA) (100 V for 60 min). Membranes were blocked with 5% skim milk for 1 h, then incubated with primary antibodies overnight at 4 ℃. Further incubation with HRP-conjugated secondary antibodies was conducted for 1 h at room temperature. Immunoreactivity was detected using Amersham™ ECL Western blotting detection reagent. Immunoblots were scanned with the Amersham Imager 600 RGB (GE Health, Chicago, USA), while quantification was performed using ImageJ.

### mCherry-GFP-LC3 transfection

SH-SY5Y cells were seeded on a 96-well plate coated with MaxGel at a density of 5000 cells/well in the indicated growth medium at the time of transfection. Transfection was performed with Lipofectamine 2000 (Invitrogen) in SH-SY5Y cells according to the manufacturer’s protocol. FUW mCherry-GFP-LC3 was a gift from Anne Brunet (Addgene, 110,060; http://n2t.net/addgene:110060; RRID: Addgene_110060) and was used for the quantification of autophagic flux [29]. Cells were transfected with the plasmid expressing mCherry-GFP-LC3 using Lipofectamine 2000 for 6 h at 37 ℃. The medium was replaced with a fresh complete culture medium, and the cells were incubated for another 24 h. Cells were then fixed and immediately visualized by confocal microscopy. A number of mCherry- and mCherry-GPF-positive puncta in each well were counted, with a minimum of 20 cells analyzed per group.

### shRNA transfection

SH-SY5Y cells were transfected with plasmids encoding shRNA against human SIRT1 (Santa Cruz) using Lipofectamine 2000, according to the manufacturer’s protocol. Negative control shRNA was used as a control to test for any possible effect of the construct on the cells. The neurite outgrowth was observed using the IncuCyte ZOOM live-cell imaging system.

### Immunofluorescence assay

Primary hippocampal neurons were cultured on coverslips coated with poly-D-lysine at a final concentration of 5 × 10^4^ cells/well. Neurons were cultured for 7 days (DIV7) for neurite outgrowth assays and 14 days (DIV14) for spine density assays. After treatment, cells were washed with 1 × PBS, followed by fixation for 15 min at room temperature with 4% paraformaldehyde in PBS. Following fixation, cells were permeabilized and blocked with 0.3% of Triton X-100 and 5% of donkey serum in PBS for 1 h. Thereafter, cells were incubated overnight with the primary antibody at 4 ℃ and then incubated with Alexa-conjugated secondary antibodies (1:400, Alexa Fluor 488 or Alexa Fluor 594, Invitrogen) in 1% donkey serum (Sigma-Aldrich) in PBS at room temperature for 1 h. Following washes, coverslips for conventional imaging were inverted onto glass microscope slides with Fluoromount-G™ Mounting Medium with DAPI (Thermo Fisher Scientific). Confocal images were acquired with a DMI6500B laser scanning confocal microscope using a 63 × oil objective. Quantification was performed using ImageJ Software.

### Statistical analysis

GraphPad Prism 7.0 Software (GraphPad Software, Inc., La Jolla, CA, USA) was utilized for statistical analyses. Differences between the two groups were determined using a Student’s *t* test. One-way or two-way ANOVAs followed by Tukey’s post hoc tests were used for multiple comparisons for more than two groups. Survival analyses were performed using the log-rank test. All *p* values < 0.05 were considered to be significant. Statistical significance is indicated by *p < 0.05, **p < 0.01, ***p < 0.001, or ns = not significant.

## Results

### CBD induces neuronal autophagy

**Fig. 1:**
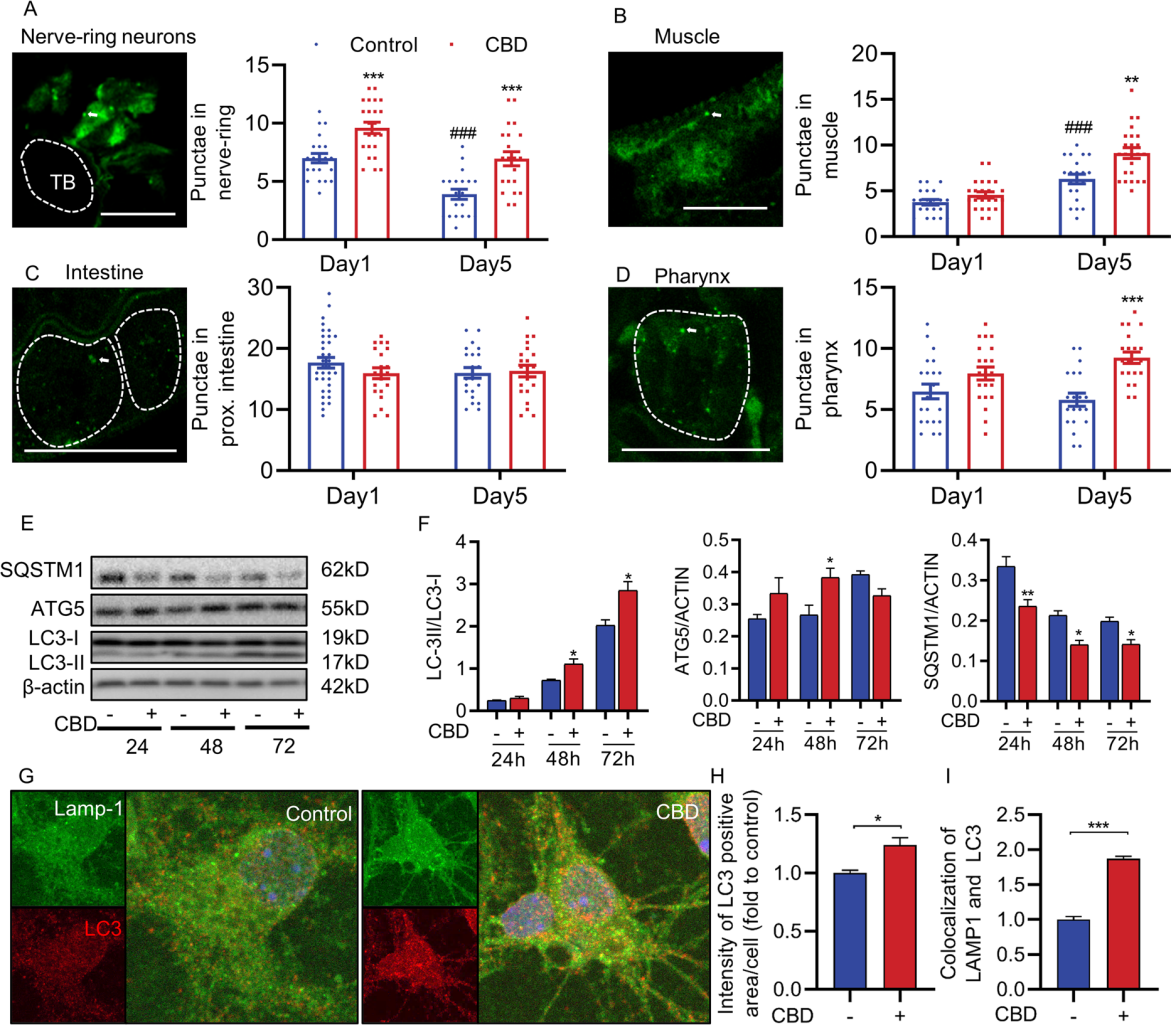
CBD induces autophagy in *C. elegans* and neuronal cells. **A** Adult transgenic animals expressing *rgef-1p::gfp::lgg-1*, imaged at day 1 and day 5 of adulthood at 20 ℃. GFP::LGG-1 punctae were counted in control and CBD animals on day 1 and day 5 of adulthood in nerve-ring neurons (day 1: control, *n* = 21; CBD, *n* = 23; day 5: control, *n* = 20; CBD, *n* = 21 animals). **B**–**D** Adult transgenic animals expressing *lgg-1p::gfp::lgg-1*, imaged at day 1 and day 5 of adulthood at 20 ℃. GFP::LGG-1 punctae were counted in control and CBD animals on day 1 and day 5 of adulthood in body-wall muscle (day 1: control, *n* = 21; CBD, *n* = 23; day 5: control, *n* = 20; CBD, *n* = 23), intestinal cells (day 1: control, *n* = 34; CBD, *n* = 21; day 5: control, *n* = 22; CBD, *n* = 22 animals), and terminal pharyngeal bulb (day 1: control, *n* = 21; CBD, *n* = 21; day 5: control, *n* = 20; CBD, *n* = 20 animals). Values are mean ± SEM. *Compared with control group; #Compared with control group in day 1. **p ≤ 0.01, *** *p* ≤ 0.001, ### *p* ≤ 0.001 by unpaired *t* test. TB, terminal bulb. Scale bar = 25 µm. **E** Western blot analysis results showing LC3-II:LC3-I ratio, and SQSTM1 and ATG5 levels normalized to actin in neuroblastoma SH-SY5Y cells at the indicated time of CBD treatment. **F** The Western blot analysis results of LC3-II:LC3-I ratio and SQSTM1 and ATG5 levels normalized to actin in SH-SY5Y cells treated with or without CBD (*n* = 3 biological replicates). *Compared with control group. *p ≤ 0.05, **p ≤ 0.01 by unpaired *t* test. **G** Immunofluorescence double-labeled staining for co-localization of LC3 with LAMP1 treated with or without CBD for 48 h in primary hippocampal neurons (green: LAMP1, red: LC3). Cell nucleus was stained with DAPI (blue). Rapamycin was used as a positive control. Scale bar = 25 µm. **H** Quantification of intensity of LC3 positive area as observed in the immunofluorescence images. *n* > 10 cells per group. **I** Quantification of co-localization of LC3 and LAMP1 observed in the immunofluorescence images. *n* > 10 cells per group. Values are mean ± SEM. *p ≤ 0.05, *** *p* ≤ 0.001 by unpaired *t* test

Autophagy eliminates defective cellular molecules via lysosome-mediated degradation during aging. Compromised autophagy is a hallmark of aging [30]. We measured autophagy during *C. elegans* aging with or without CBD treatment. The binding of the autophagy receptors is facilitated by ATG8/LC3 family proteins. LGG-1 is the *C. elegans* ortholog of mammalian LC3 and yeast ATG8. We first examined the induction of autophagy after CBD treatment using transgenic worms expressing GFP-tagged LGG-1, an autophagy reporter, visualizes autophagic structures as fluorescent punctae [31]. The number of APs was measured by counting GFP::LGG-1 positive punctae in nerve-ring neurons, intestine, body-wall muscle, and the pharynx [32]. During normal aging without CBD treatment, the nerve-ring neuron was the only tissue among all measured tissues, significantly reducing APs from day 1 to day 5 (− 44.28%, *p* < 0.001). One-day CBD treatment significantly increased APs in nerve-ring neurons (34.14%, *p* < 0.001) but not any other tissues compared with the controls (Fig. 1AD). Furthermore, a 5-day CBD treatment significantly increased APs in nerve-ring neurons, body-wall muscle, and pharynx (78.25%, 44.92%, 59.48%, all *p* < 0.01), but not intestine, compared with controls (Fig. 1AD).

To further validate the neuronal effect, we treated SH-SY5Y neurons with CBD. The result showed that CBD modulated the autophagy in SH-SY5Y neurons in a time-dependent manner. It is that CBD for 48 h increased LC3-II/LC3-I (53.19%, *p* < 0.05, Fig. 1E and F) but decreased SQSTM1 (− 34.22%, *p* < 0.01, Fig. 1E and F), indicating that CBD induces autophagy in SH-SY5Y neurons. Similarly, the mouse primary hippocampal neurons showed activated autophagy after CBD treatment (Fig. 1G). Hippocampal neurons treated with CBD showed increased LC3 intensity compared with the control untreated group (24.1%, *p* < 0.05, Fig. 1H). In addition, CBD treatment led to increased co-localization of LC3 and LAMP1, lysosomal membrane, in primary hippocampal neurons (86.9%, *p* < 0.001, Fig. 1I). These results based on in vivo and in vitro studies indicate that CBD induces neuronal autophagy.

### CBD promotes autophagic flux in neurons

**Fig. 2:**
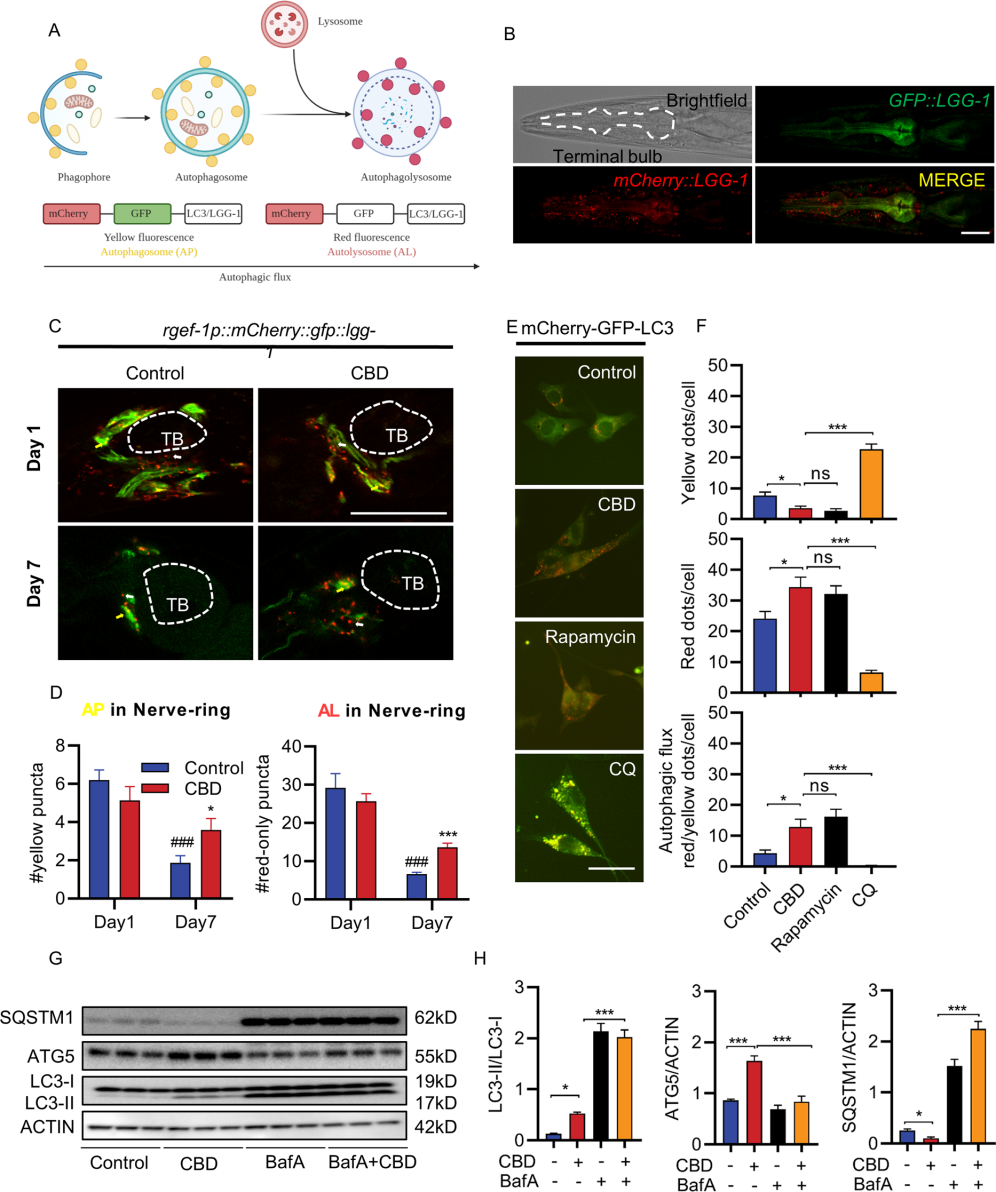
CBD induces autophagic activity in nerve-ring neurons in *C. elegans* and neuronal cells. **A** Schematic representation of *mCherry::GFP::lgg-1* fluorescence states in the autophagy pathway. **B** Terminal pharyngeal bulb expression of *mCherry::GFP::lgg-1* in a wild-type (WT) animal at day 1 of adulthood. Scale bar = 20 µm. **C** Adult transgenic WT animals expressing *rgef-1p::mCherry::GFP::lgg-1* in nerve-ring neurons imaged at day 1 and day 7 of adulthood with or without CBD. Yellow arrows denote the autophagosome (AP) punctae with mCherry/GFP signals. White arrows denote the autolysosome (AL) punctae with only mCherry signals. Scale bar = 25 µm. **D** Quantification of autophagosomes (APs) and autolysosomes (ALs) in adult day 1 and day 7 animals expressing *lgg-1p::mCherry::GFP::lgg-1* in pharynx and *rgef-1p::mCherry::GFP::lgg-1* in nerve-ring neurons. Worms were treated or not treated with CBD from egg. Values are mean ± SEM of ≥ 15 animals combined from three independent experiments. TB, terminal bulb. *Compared with control group; #compared with control group in day 1. *p ≤ 0.05, *** *p* ≤ 0.001, ### *p* ≤ 0.001 by unpaired *t* test. **E** Representative images of SH-SY5Y cells transfected with mCherry-GFP-LC3 for 48 h, followed by CBD treatment. Chloroquine (CQ) was used as negative control as it blocks the binding of autophagosomes to lysosomes by altering the acidic environment of lysosomes. Rapamycin was used as positive control as it induces autophagic flux. Scale bar = 25 µm. **F** Quantification of autophagic flux from experiment shown in **E**. The numbers of autophagosomes (yellow dots) and autolysosomes (red dots) were manually counted. Autophagic flux is estimated as the ratio between red and yellow dots. *n* > 20 cells per treatment. **G** Representative Western blots showing the LC3-II:LC3-I ratio and the expression of SQSTM1 and ATG5 of SH-SY5Y cells treated with or without CBD for 48 h and/or with bafilomycin A (BafA), an inhibitor of lysosomal acidification, at 10 nM for 6 h (*n* = 3 biological replicates). **H** The Western blot analysis results of LC3-II:LC3-I ratio and SQSTM1 and ATG5 levels normalized to actin in SH-SY5Y cells treated with or without CBD for 48 h and/or BafA for 6 h

Autophagic flux is an indication of autophagy completion, including autophagosome and autophagolysosome formation. Autophagic flux was detected by dual-florescent mCherr::GFP::LGG-1 reporter monitors for APs and autolysosome (AL) formation [32]. GFP and mCherry have different sensitivities to the acidic pH of the lysosome, which enables ALs (mCherry-only puncta) to be distinguished from APs (GFP and mCherry positive puncta, which appear yellow) (Fig. 2A). We used transgenic worms expressing mCherry::GFP::LGG-1 from a pan-neuronal *rgef-1* promoter to specifically visualize autophagic flux in neurons (Fig. 2B). Interestingly, the nerve-ring neurons showed an age-associated decrease in the number of APs (− 69.76%, *p* < 0.001) and ALs (− 77.31%, *p* < 0.001) when assessed with the *mCherry::GFP::LGG-1* reporter (Fig. 2C and D). However, more APs and ALs were observed in the nerve-ring neurons after CBD treatment compared with control worms on day 7 (AP: 91.1%, *p* < 0.05; AL: 106.34%, *p* < 0.001; Fig. 2C and D).

To further validate autophagic flux in neurons, we monitored SH-SY5Y cells transfected with *mCherry-GFP-LC3* plasmid using the IncuCyte live imaging system, similar to the experiment performed above in *C. elegans*. To demonstrate the capacity of this system to monitor autophagic flux, we performed two control treatments. Rapamycin treatment was used as a positive control; more red puncta are observed in the presence of rapamycin because of the increased fusion of autophagosomes with lysosomes. Meanwhile, inhibition of autophagic flux using chloroquine (CQ) treatment, which blocks the binding of autophagosomes to lysosomes, showed more yellow puncta (Fig. 2E). Similar to the effect of rapamycin treatment, CBD treatment led to the observation of more red puncta than yellow puncta, suggesting that CBD promotes autophagic flux (200.94%, *p* < 0.05, Fig. 2F). These findings indicate that autophagic flux decreased during aging in the neurons of *C. elegans*. However, CBD promotes autophagic flux in neurons.

### CBD-mediated longevity requires autophagy integrity

**Fig. 3:**
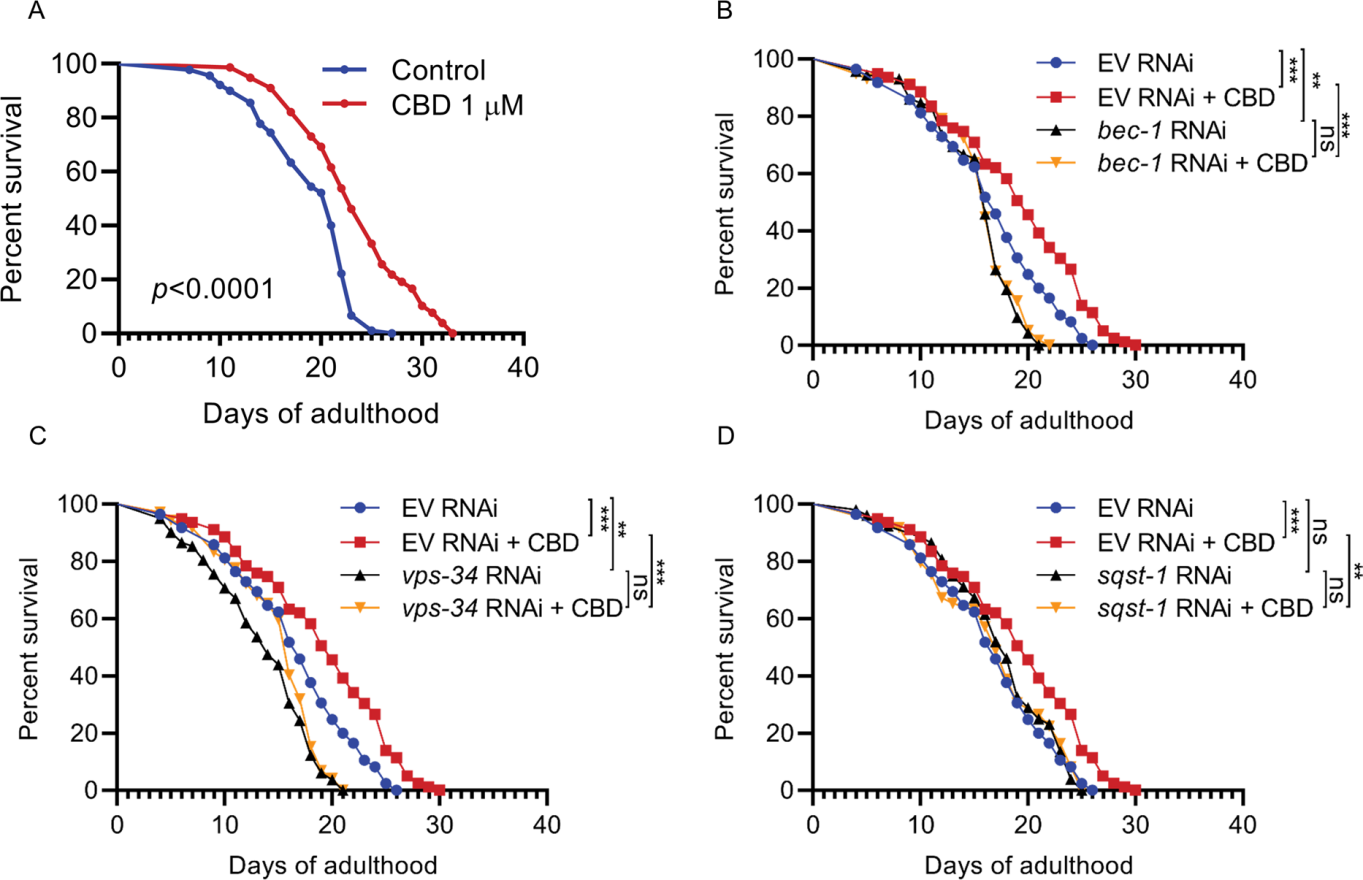
Autophagy genes are required for CBD-mediated lifespan extension. **A** Lifespan of wild-type worms (N2) treated with 1 μM or vehicle treatment at 20℃. *p* values compared with control calculated using log-rank test. **B**, **C** Lifespan of worms fed with bacteria containing empty vector (EV) or vectors for RNAi of autophagy genes (*vps-34*, *bec-1*, and *sqst-1*), treated with CBD or untreated. *p ≤ 0.05, **p ≤ 0.01, *** *p* ≤ 0.001 by log-rank test. Data are representative of at least three independent experiments

The rate of autophagic flux decreases with advancing age and a lifespan extension in *C. elegans* [33, 34]. The autophagy genes are the basic requirement for autophagic flux. In worms, *bec-1* is the ortholog of the yeast and mammalian autophagy proteins Atg6/Vps30 and Beclin 1, which interacts with the class III PI3 kinase *vps-34*, an essential protein required for autophagy, membrane trafficking, and endocytosis [35]. Autophagy can degrade cargos with the help of selective autophagy receptors such as p62/SQSTM1 [36]. The lifespan was significantly increased in C. *elegans* treated with 1 μM (*p* < 0.001, Fig. 3A, Table S2) and also 5 and 10 μM CBD (both *p* < 0.01, Fig. S1A and B). To further understand the effects of CBD on the crosstalk between autophagy and longevity, we knocked down autophagy genes *sqst-1*, *vps-34*, and *bec-1* by RNAi and assessed lifespan in these experimental groups. We found that *bec-1* or *vps-34* RNAi treatment significantly shortened lifespan compared with the control (empty vector, EV) RNAi group (all *p* < 0.01). Furthermore, CBD treatment failed to extend lifespan in *bec-1* and *vps-34* RNAi groups (Fig. 3B and C). When we performed *sqst-1* RNAi, this did not affect lifespan compared with the EV RNAi group; however, CBD failed to extend lifespan in the *sqst-1* RNAi group (Fig. 3D). These results suggest that *bec-1* and *vps-34*, which induce autophagic vesicle nucleation, were required for a normal lifespan while *sqst-1* was not. All essential autophagy genes (*bec-1*, *vps-34*, and *sqst-1*) were required for the lifespan increase mediated by CBD treatment.

### CBD improves healthspan and neuronal morphology associated with aging in C. elegans

**Fig. 4:**
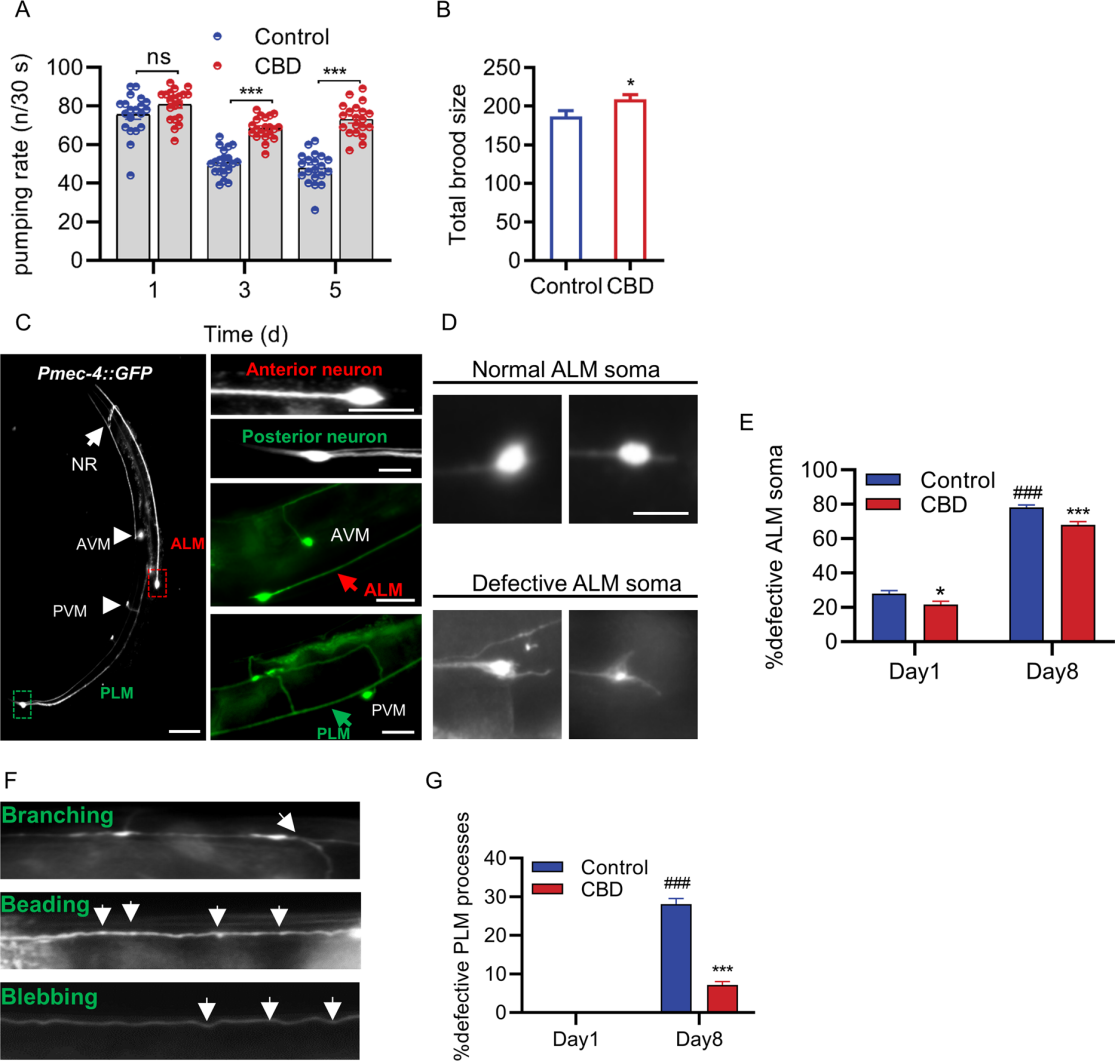
CBD improves healthspan and neuronal health during aging in *C. elegans*. **A** Pharyngeal pumping rate at days 1, 3, and 5 of adulthood in worms treated with 1 µM CBD or vehicle, *n* = 20–25 animals per group. Values are mean ± SEM. **p ≤ 0.01, *** *p* ≤ 0.001 using an unpaired *t* test. **B** Comparison of total brood size between groups treated with 1 µM CBD or vehicle, *n* = 20–25 animals per group. Values are mean ± SEM. *p ≤ 0.05 using an unpaired *t* test. **C** Confocal fluorescent images with GFP-visualized touch receptor neurons in day 2 adults. The strain QH3135*zdls5* [P_*mec-4*_::GFP] contains an integrated transgene expressing GFP in ALM (L/R) (red arrowhead) and PLM (L/R) (green arrowhead) neuron pairs, and in AVM and PVM (arrowhead) neurons. The dashed boxes indicate the regions in the soma of ALM neuron (red) and PLM neuron (green). Scale bar = 50 µm (left panel), scale bar = 10 µm (right panel). **D** Representative images of normal ALM soma and defective ALM soma. Scale bar = 10 µm. **E** quantification of ALM neurons with defective soma in WT worms treated with or without CBD in day 1 and day 8. *Compared with control group; #compared with control group in day 1. *p ≤ 0.05, *** *p* ≤ 0.001, ### *p* ≤ 0.001 by unpaired *t* test. **F** Representative confocal fluorescent images of age-dependent defects in PLM processes. Arrows mark neurite branching, beading, and blebbing. Scale bar = 25 µm. **G** Quantification of the PLM process defects in WT worms treated with or without CBD in day 1 and day 8. At least 30 neurons were scored per time point. Values are mean ± SEM of three independent experiments. *Compared with control group; #compared with control group in day 1. *** *p* ≤ 0.001, ### *p* ≤ 0.001 by unpaired *t* test

Aging can lead to poor health, movement retardation, and neuronal degeneration [37]. The life quality of aging is equally important as living longer. Whether or not CBD improves healthspan is not known. The healthspan indicator used in this study includes pharyngeal pumping rate, hermaphrodite reproductive capacity, and locomotion in *C. elegans* as per previous studies [38, 39]. The declines of these parameters cause a decline in survival probability [40]. CBD treatment significantly increased the pumping rate on days 3 and 5 compared to the controls (day 3: 34.95%, *p* < 0.001; day 5: 53.10%, *p* < 0.001; Fig. 4A). Substantially, more eggs were laid on day 5 of *C. elegans* treated with CBD compared with controls (266.67%, *p* < 0.01, Fig. S1C), although there was no difference between the two groups on day 1 (Fig. S1C). Total brood size (the number of progeny) was increased after treatment with CBD (11.89%, *p* < 0.05, Fig. 4B). Body movements as the body bend in 20 s were declined with increasing age in *C. elegans* measured on days 1, 3, 5, 7, 9, and 11 of adulthood (day 3: 0.3%; day 5: 7.6%; day 7: 27.20%; day 9: 36.77%; day 11: 45.89%, compared with day 1; Fig. S1D). Overall, CBD treatment increased body bends over 11 days, with day 7 showing a significant increase (24.72%, *p* < 0.05, Fig. S1D).

A feature of neuronal aging is morphological changes, which are progressive changes associated with brain function alteration [41–43]. The age-associated neuronal morphological changes are frequently studied in *C. elegans* including branching, beading, and blebbing in the anterior and posterior touch receptor neurons (ALM, PLM), which are also referred to as “defective neurons.” Using a transgenic line expressing a TRN-specific *mec-4p*::*gfp* transgene, we measured the morphological changes in ALM and PLM neurons in young and aged *C. elegans* (Fig. 4C,D, and F). In ALM neurons, *C. elegans* significantly increased the number of irregularly shaped soma on day 8 compared with day 1 (179.04%, *p* < 0.001, Fig. 4E). CBD treatment significantly reduced these changes on both days 1 and 8 compared with controls (day 1: 22.36%, *p* < 0.05; day 8: 12.97%, *p* < 0.001; Fig. 4E). In PLM neurons, *C. elegans* significantly increased the branching, beading, and blebbing on day 8 compared to day 1 (Fig. 4G). CBD treatment for 8 days significantly reduced the proportion of defective PLM processes (65.09%, *p* < 0.001, Fig. 4G). The results suggest that CBD slows down neuronal aging.

### CBD requires autophagy to protect age-associated neuronal morphological changes

**Fig. 5:**
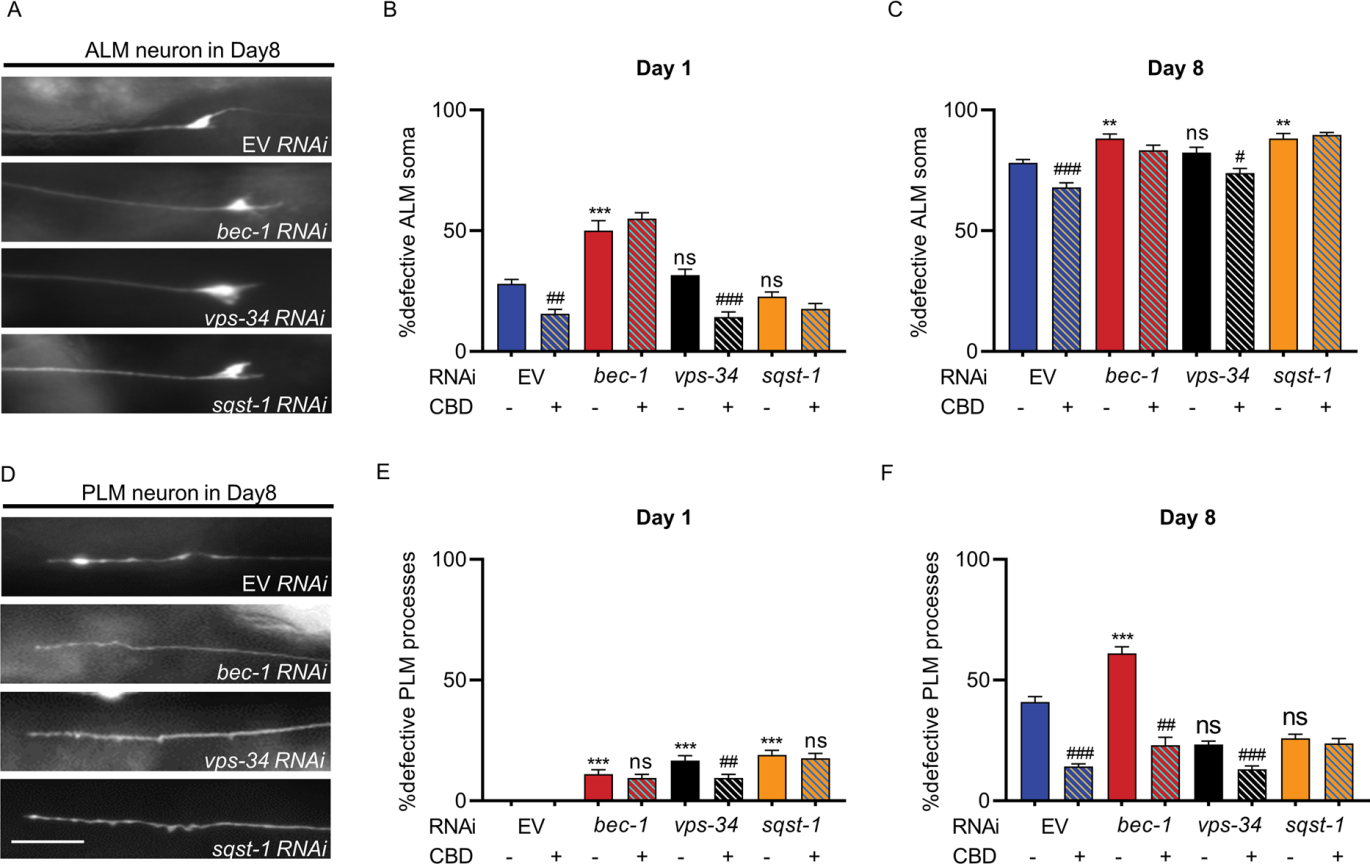
CBD affects age-associated morphological changes in ALM and PLM neurons via autophagy pathway. **A** Representative images of ALM soma defective in worms fed with EV and autophagy genes (*bec-1*, *vps-34*, and *sqst-1*) RNAi in adult day 8. **B**, **C** Quantification of ALM neurons with defective soma in WT worm strains fed with bacteria containing EV or vectors for RNAi knockdown of autophagy genes (*bec-1*, *vps-34*, and *sqst-1*) RNAi and treated with or without CBD in adult day 1 and day 8. **D** Representative images of PLM process defective in worms fed with EV and autophagy genes (*bec-1*, *vps-34*, and *sqst-1*) RNAi in adult day 8. **E**, **F** Quantification of the PLM process defects in worms fed with EV and RNAi constructs for knockdown of autophagy genes (*bec-1*, *vps-34*, and *sqst-1*) and treated with CBD or untreated at day 1and day 8. Error bars are SEs of proportions. At least 30 neurons were scored per time point. *Compared with EV control; #compared with same RNAi without CBD. ns, not significant. **p ≤ 0.01, *** *p* ≤ 0.001, # ≤ 0.05, ## ≤ 0.01, ### ≤ 0.001

Since autophagy genes (*bec-1*, *vps-34*, and *sqst-1*) were required for CBD-mediated longevity, we investigated whether autophagy processes were involved in regulating age-associated morphological changes. In young (day 1 adult) worms in which key autophagy genes (*bec-1*, *vps-34*, and *sqst-1*) were knocked down with RNAi, no significant changes in ALM defects were observed compared with the EV group, except for the *bec-1* RNAi cohort, which showed a significant increase in ALM defects (78.57%, *p* < 0.001, Fig. 5B). In old (day 8 adult) worms, knockdown of *bec-1*(12.93%, *p* < 0.01) and *sqst-1* (12.93%, *p* < 0.01) by RNAi led to a significant increase in ALM defects compared with controls (Fig. 5A  and C). On day 8, 40.9% of the control RNAi (EV) group showed a defective PLM process, compared with worms on day 1 (Fig. 5D,E and F). Similar to effects on ALM, the *bec-1* RNAi treatment showed higher defective PLM neurons on day 1 (11.11%, *p* < 0.001) and day 8 (49.38%, *p* < 0.001) (Fig. 5D). These data indicate that *bec-1* RNAi leads to more defective in ALM and PLM neurons, while *sqst-1* increases the percentage of defective ALM soma, but not PLM on day 8.

We then tested whether autophagy genes might modulate ALM and PLM during aging after CBD treatment. RNAi EV + no CBD treatment control shows age-related ALM/PLM defects. RNAi EV + CBD treatment shows a lower proportion of ALM/PLM defects on day 8, showing that CBD treatment protects from neuronal aging phenotypes (ALM: 12.97%, *p* < 0.001; PLM: 65.07%, *p* < 0.001; Fig. 5C and F). Furthermore, CBD treatment in experimental groups in which *bec-1* and *sqst-1* were knocked down with RNAi showed similar levels of ALM defects compared with RNAi EV + CBD group on day 1 and day 8. However, CBD treatment showed a lower proportion of both ALM and PLM defects in *vps-34* RNAi group in day 1 (ALM: 54.75%, *p* < 0.001; PLM: 42.85%, *p* < 0.01) and day 8 (ALM: 10.24%, *p* < 0.05; PLM: 44.10%, *p* < 0.001; Fig. 5B,C,D, and F) suggesting that *vps-34* is not required for CBD-mediated effects on neuronal aging. These data suggest that *bec-1* and *sqst-1* were required for the action of CBD on neuronal aging.

### CBD requires sir-2.1 to improve lifespan, autophagy, and neuronal aging

**Fig. 6:**
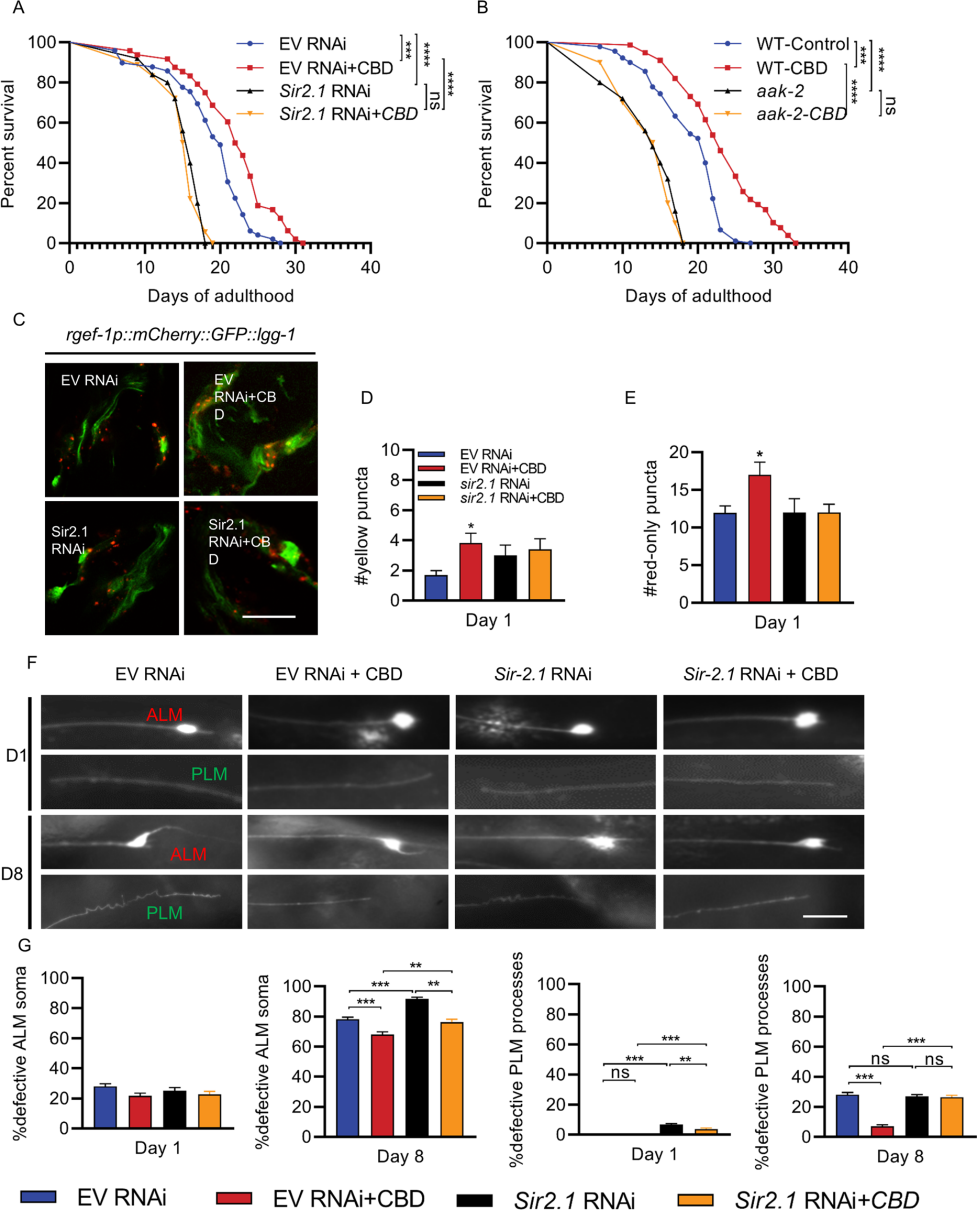
CBD regulates progressive touch neuron defects through *sir-2.1*. **A** Lifespan analysis of WT worm strains at 20 ℃ fed with empty vector RNAi bacteria (EV) and *sir-2.1* RNAi, treated with CBD or untreated. *p* values were determined using a log-rank test. **B** Lifespan analysis of WT strains and *aak-2(ok524)* mutant strains at 20 ℃ treated with CBD or untreated. *p* values by log-rank test. **C** Fluorescence images of autophagic flux in adult day 1 expressing *rgef-1p::mCherry::GFP::lgg-1* in nerve-ring neurons. Worms fed with EV and sir-2.1 RNAi are treated with or without CBD. Scale bar = 25 μm. **D**, **E** Quantification of autophagosome (AP) punctae with mCherry/GFP signals and autolysosome (AL) punctae with only mCherry signals. Values are mean ± SEM of ≥ 15 animals combined from three independent experiments. *p ≤ 0.05 compared with control group by one-way ANOVA. **F** Fluorescence images of age-dependent defects of the ALM soma and PLM processes in worms fed with control EV or *sir-2.1* RNAi and treated with CBD or untreated. Scale bar = 25 µm. **D** Quantification of ALM neurons with defective soma in worms fed with EV and *sir-2.1* RNAi, treated with CBD or untreated, in day 1 and 8 adults. **G** Quantification of the PLM process defective in worms fed with EV and *sir-2.1* RNAi and treated with or without CBD in day 1 and 8 adults. Error bars are SEs of proportions. At least 30 neurons were scored per time point. ns, not significant. *p ≤ 0.05, **p ≤ 0.01, *** *p* ≤ 0.001

SIRT1, a class III histone deacetylase, links to the extension of lifespan and age-related cellular mechanisms [44]. The ortholog of SIRT1 in *C. elegans* is *sir-2.1* [45]. To determine whether CBD regulates lifespan extension in *C. elegans* via the *sir-2.1/aak-2* pathway, we performed a lifespan assay in worms in which *sir-2.1* had been knocked down via RNAi, or in *aak-2* mutant animals. We found that the lifespan of the *sir-2.1* RNAi group was significantly shorter than the control RNAi (EV) group (*p* < 0.001, Fig. 6A). We also found that the *aak-2* mutant was significantly short-lived compared with wild-type controls (*p* < 0.001, Fig. 6B). CBD treatment did not affect the shortened lifespan of *sir-2.1* (RNAi) or *aak-2* mutant groups (Fig. 6A and B). The results suggest that *sir-2.1* and *aak-2* are required for CBD-induced longevity.

Transgenic overexpression or pharmacological activation of SIRT1 stimulates autophagic flux in both worms and human cells [9]. We examined autophagic flux in nerve-ring neurons expressing *mCherry::GFP::LGG-1* of the worms with or without *sir-2.1* RNAi (Fig. 6C). CBD increased the number of APs and ALs, but the effect disappeared in the *sir-2.1* RNAi worms compared to controls (Fig. 6D and E).

We examined whether *sir-2.1* is required for CBD-dependent effects on age-associated neuronal morphological changes. We examined ALM and PLM neurons of worms with or without *sir-2.1* RNAi treatment from the embryo/egg stage and then treated with or without CBD at day 1 and day 8 (Fig. 6F). There were age-dependent defects in both ALM and PLM neurons in wild-type worms. Compared with the controls, *sir-2.1* RNAi significantly increased ALM soma defects at day 8, but not PLM (17.33%, *p* < 0.001, Fig. 6G). We next examined whether CBD affects neuronal aging through *sir-2.1*. We found that *sir-2.1* RNAi + CBD treatment shows a higher proportion of ALM/PLM defects at day 8 (ALM: 12.04%, *p* < 0.01; PLM: 270.6%, *p* < 0.001; Fig. 6G), compared with the EV RNAi + CBD group. In addition, CBD-mediated age-associated morphological changes in PLM neurons were significantly abrogated by RNAi of *sir-2.1* on day 8 (Fig. 6G).

Overall, these results suggest that the protective role of CBD in *C. elegans* lifespan, autophagy, and neuronal aging involves *sir-2.1*. A deficiency of *sir-2.1* results in shortened lifespan, impaired autophagic flux, and triggered early neuronal aging.

### SIRT1 is required for CBD-enhanced neurite outgrowth and spine density

**Fig. 7:**
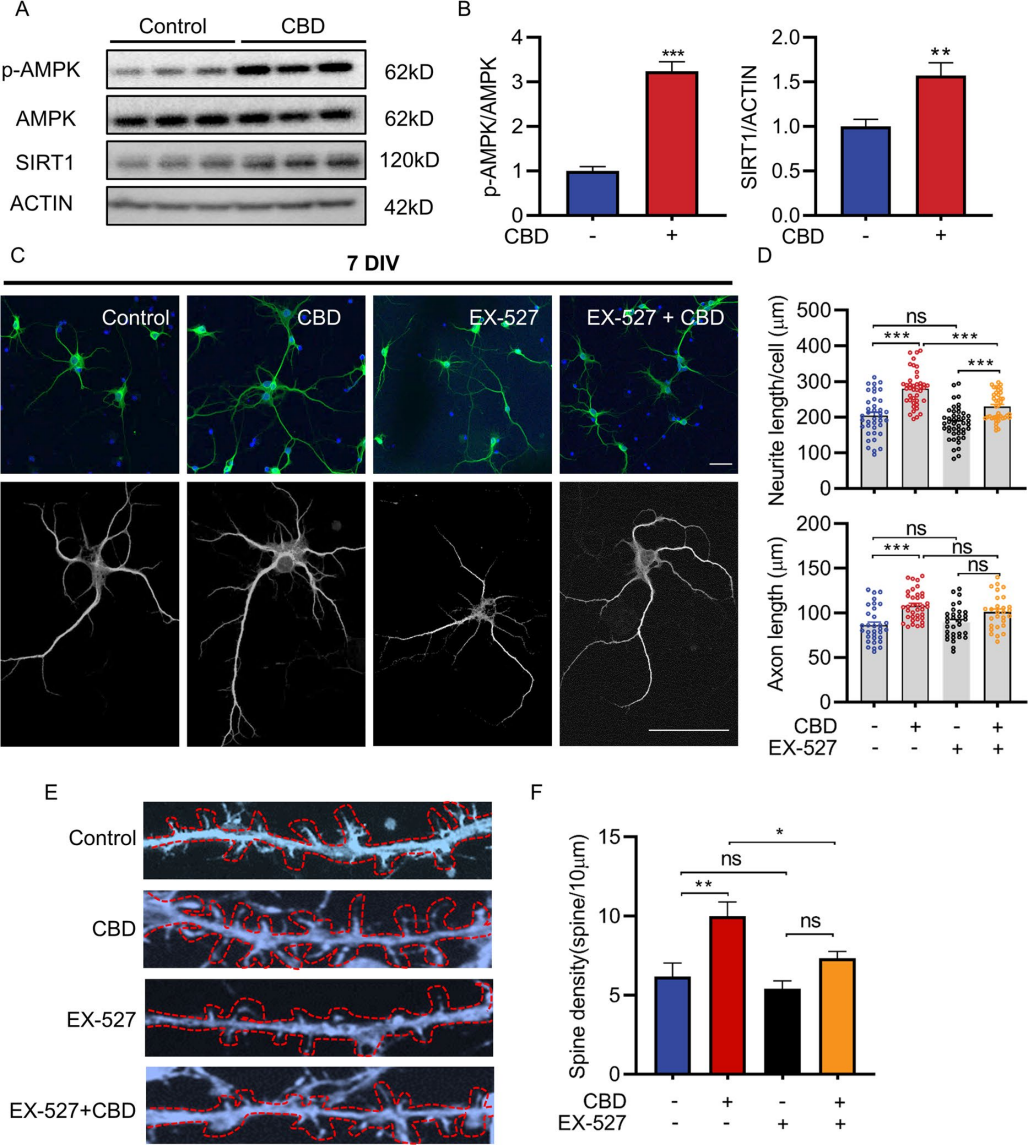
CBD enhances neurite outgrowth and increases dendritic spine density via SIRT1. **A** Representative Western blots showing the expression of SIRT1 and p-AMPK/AMPK in SH-SY5Y cells treated with or without CBD for 48 h. **B** Western blot analysis for SIRT1 and p-AMPK/AMPK in SH-SY5Y cells treated with or without CBD for 48 h. Western blots shown are representative of *n* = 3 biological replicates. **C** Representative images of primary hippocampal neurons at DIV7 pre-treated with EX-527, a selective inhibitor of SIRT1, followed the CBD treatment for 24 h. Neurons were then fixed and stained for MAP2 (green) and DAPI (blue). Scale bar = 50 µm. **D** Quantification of neurite length in four groups (control, 117 neurites, 39 neurons; CBD, 133 neurites, 41 neurons; EX-527, 113 neurites, 45 neurons; EX-527 + CBD, 114 neurites, 40 neurons). Quantification of axon length in four groups (control, 32 axons; CBD, 37 axons; EX-527, 32 axons; EX-527 + CBD, 28 axons). **E** Representative high magnification images of dendrites immunostained with phalloidin-actin after CBD and/or EX-527 treatment. Scale bar = 10 µm. **F** Quantification of dendritic spine density. *n* > 5 neurons per treatment. Values are mean ± SEM. *p ≤ 0.05, **p ≤ 0.01, *** *p* ≤ 0.001 using a one-way ANOVA

To further validate the neuronal effect, we examined if knocking down SIRT1 alters the neurite outgrowth induced by CBD in SH-SY5Y neurons. Compared with the control group, CBD treatment upregulated the expression of SIRT1 (57.1%, *p* < 0.01) and p-AMPK/AMPK (224.1%, *p* < 0.001, Fig. 7A and B). We measured the neurite length in real time and showed that the neurite outgrowth of CBD treatment was reduced when SIRT1 levels were downregulated by shRNA-SIRT1 (27.04%, *p* < 0.001, Fig. S2A and B). We also confirmed these findings in primary hippocampal neurons with or without CBD treatment. Hippocampal neurons at 7 days in vitro (DIV) pre-treated with EX-527 for a half-hour, a selective inhibitor of SIRT1, were then exposed to CBD for 48 h. Neurite length and axon length were visualized by staining these neurons with a MAP2 antibody and quantified by ImageJ (Fig. 7C). We observed increased total neurite length (37.19%, *p* < 0.001, Fig. 7D) as well as mean neurite length after CBD treatment (16.21%, *p* < 0.001, Fig. S2C). Furthermore, the axon length in the CBD group was also longer than in the control (no CBD/EX-527 treatment) group (25.18%, *p* < 0.001, Fig. 7D). Conversely, a SIRT1 inhibitor EX-527 attenuated CBD’s effect on neurite length while not on axon length. Furthermore, the control group and CBD group showed higher distribution in 3 neurites and 4 neurites per neuron. However, the higher distribution of neurite number per neuron was changed to 2 and 3 in the EX-527 group treated and untreated with CBD (Fig. S2D). We also examined spine density in CBD- and/or EX-527-treated neurons. Hippocampal neurons were treated with EX-527 at DIV11 and then exposed to CBD for 3 days. The neurons were fixed at DIV14 and the morphology of the spines was visualized by confocal microscopy (Fig. 7E). Treatment with CBD significantly increased dendritic spine density compared with the control group (62.15%, *p* < 0.01, Fig. 7F). Neurons treated with EX-527 blocked CBD’s effect to increase dendritic density (Fig. 7F). Together, these results indicate that CBD can enhance neurite length and increase spine density; however, the effects were abolished by blocking SIRT1.

## Discussion

In the present study, we have demonstrated the following: (1) treatment of CBD induces neuronal autophagy both in *C. elegans* and neuronal cells; (2) CBD treatment increases autophagic activity during aging and rescues the decrease in autophagic activity in nerve-ring neurons; (3) autophagy genes are required in CBD-mediated longevity; (4) CBD improves healthspan and neuronal morphology associated with aging in *C. elegans*; (5) *Bec-1* and *sqst-1* are required for the action of CBD on neuronal aging, not *vps-34*; (6) CBD regulates autophagy and neuronal aging through *sir-2.1*; (7) SIRT1 is required for CBD-enhanced neurite outgrowth and spine density.

### *CBD induces neuronal autophagy *in vivo* and *in vitro* during aging*

Autophagy is active in the intestine, muscle, pharynx, and neurons of aging *C. elegans*, whereas autophagic flux appears to decrease with age [32]. Recent studies report that the induction of autophagy/mitophagy by CBD in neuronal cells [20] and glioma cells [46] can elicit neuroprotective or antitumor effects. However, the tissue specificity of CBD in the induction of autophagy is not known. We found that neurons had the earliest response to CBD treatment (1 day) compared with other tissues which require a longer duration of treatment (5 days) in *C. elegans*. The different responses observed here may be due to the tissue-specific variations in basal autophagy change during aging. Accumulating evidence suggests that suppression of autophagic activity is one of the signatures of aging [47]. Our study showed that CBD rescued the decrease in autophagic activity in nerve-ring neurons, which may contribute to its anti-aging effect in neuronal aging.

### Autophagy genes are required in CBD-mediated longevity

Recent studies in *C. elegans* have provided further evidence for the role of autophagy in longevity. Tissue-specific overexpression of a single autophagy gene (i.e., *unc-51*, *bec-1*, *pink-1*, *sqst-1*) or autophagy stimulation in select tissues has beneficial effects on lifespan [48]. In this context, the autophagy induction property of CBD may contribute to lifespan extension. To investigate the role of autophagy genes in CBD-mediated longevity of *C. elegans*, we knocked down *bec-1*, *vps-34*, and *sqst-1*, the key genes of the autophagy pathway, using RNAi and examined changes in lifespan. Knocking down *bec-1* and *vps-34* with RNAi could potentially shorten lifespan, specifically in long-lived mutants, by blocking endocytosis [33]. In line with these findings, we found knockdown of *bec-1* and *vps-34* failed to enhance the gain in longevity mediated by CBD treatment. However, the knockdown of *sqst-1* did not affect longevity, whereas the beneficial effect on the longevity of CBD was abolished by the knockdown of the autophagy receptor *sqst-1*. Interestingly, our observation is consistent with the recent study which indicated that *sqst-1*, unlike other autophagy genes, is not broadly implicated in lifespan extension, but instead is required under specific conditions, such as heat shock [36]. Therefore, we concluded that autophagy genes (*bec-1*, *vps-34*, and *sqst-1*) contribute to the effect of CBD on longevity in *C. elegans*.

### CBD improves healthspan and delays neuronal aging in C. elegans

Although increasing evidence has shown the beneficial effects of CBD in different models of aging and age-associated disease [17–19, 49], the molecular mechanism of how CBD affects neuronal aging remains elusive. In *C. elegans*, many physiological changes at the tissue, cellular, and molecular levels occur that are closely related to the effects of aging [22]. Similar to another study in zebrafish (*Danio rerio*) [19], the effect of CBD on lifespan extension was observed in the micromolar concentration range in wild-type *C. elegans*. The most common assumption is that lifespan extension would also result in an increase in healthspan, which is a separate but equally important measure of aging compared with lifespan [39]. Our previous study shows that CBD increases lifespan in *C. elegans* [18]. The present study showed that CBD improved almost all the age-associated physiological functions, including total brood size, pumping rate, and body bends. We observed that CBD increased reproduction in early adulthood, days 1 and 5, supporting the previous study [50]. They found increased intestinal autophagy promoting reproduction study; however, our present study did not show increased intestinal autophagy after CBD treatment, suggesting that other factors besides autophagy could be involved. Furthermore, their study reported that the later intestinal autophagy could promote intestinal atrophy and yolk steatosis in later life in *C. elegans*, which was not investigated in our present study, and warrants future research.

Neuronal aging is associated with a decline in behavioral and cognitive functions of the brain [51]. Alterations in neuronal cytoarchitecture are hallmarks of aging both in the vertebrate and the invertebrate nervous systems [25]. Age-dependent defects have been observed in *C. elegans* touch receptor neurons, including loss of synaptic integrity and abnormal outgrowths [52]. In line with previous reports, we observed irregular ALM soma and defective PLM processes (i.e., beading, blebbing, and branching) during aging. These results suggest that CBD treatment may help to stabilize the neuronal structure.

### CBD protects neuronal aging through the autophagy pathway

To confirm the potential role of autophagy genes (*bec-1*, *vps-34*, and *sqst-1*) in neuronal aging, the ALM and PLM processes were determined by RNAi. Results observed in a recent study showed a delay in the development of GABAergic motor neurons and defective locomotion in *bec-1 (ok691)* homozygous mutants [53]. Indeed, we observed significant defects of ALM and PLM neurons in *bec-1* RNAi worms during aging. However, the addition of CBD did not rescue the defective neurons in the *bec-1* and *sqst-1* groups, but did in the *vps-34* RNAi group. In addition to their roles in autophagy, *vps-34* is also required for endocytosis. Knocking down *vps-34* with RNAi could potentially shorten lifespan by blocking endocytosis [33]. Therefore, CBD may have a differential role in affecting autophagy genes. It would therefore be interesting to further explore the different autophagy gene’s specific role in particular neurons in aging and neurodegenerative diseases.

### SIRT1/sir-2.1 involves in CBD-induced longevity and neuroprotection

What are the signaling pathways associated with autophagy that may be responsible for CBD-mediated anti-aging effects? We focused our attention on SIRT1, which plays an important role in the regulation of autophagy [54]. We then further investigated the role of *sir-2.1* (a *C. elegans* ortholog of SIRT1) in the longevity and neuronal aging of *C. elegans*. In combination with the insulin/IGF pathway, sir-2.1 has been reported to affect the longevity of C. *elegans* [55]. However, it has also been proposed that sir-2.1 may regulate lifespan in a *daf-16*-independent manner involving AMP-activated protein kinase (AMPK) signaling under specific conditions, such as dietary restriction [56, 57]. Our observation showed that *sir-2.1* RNAi and *aak-2(ok524)* mutant displayed a shorter lifespan compared with the control group and failed to enhance the gain in lifespan mediated by CBD treatment. Overexpression of *sir-2.1* induced autophagy and increased expression and cytoplasmic aggregation of LGG-1 (a *C. elegans* ortholog of LC3) reporter in *C. elegans* [9]. The lifespan-extending effect of dietary restriction on aging in *Drosophila* has also been reported to be *dSir2* dependent [58]. Our results showed that CBD-induced autophagic flux in the nerve-ring neurons was disrupted by the knockdown of *sir-2.1*. It will be interesting to investigate how CBD affects age-dependent decline in autophagic activity in other tissues. In addition, knockdown of *sir-2.1* using RNAi displayed ALM soma and PLM process defection similar to normal aging (EV group). It is noteworthy that these effects can be rescued by CBD treatment in ALM, not in PLM. This suggests that *sir-2.1* may be involved in CBD-induced longevity and neuroprotection which is related to the autophagy pathway.

The notion that SIRT1 controls mammalian cell growth and survival under stress is widely recognized; however, this effect has been reported as either a promoter or suppressor depending on the cell types studied, ranging from immortalized cell lines of various lineages to cancer cells and neural stem cells. A recent study showed that CBD induced autophagy in SH-SY5Y cells, which protected the cell from mitochondrial dysfunction by upregulating SIRT1 and inhibiting NF-KB and Notch pathways [59]. Our study showed that CBD increased neurite outgrowth and spine density via SIRT1. These data suggest that SIRT1 plays a pivotal role in supporting CBD-mediated neurite outgrowth.

## Conclusion

In conclusion, we report that CBD induced autophagy in vitro and in vivo and discovered the critical role of SIRT1/*sir-2.1*-elicited autophagy in mediating the anti-aging and neuroprotective effects of CBD. Our study provides evidence that CBD contributed to extending lifespan and ameliorating the deterioration of aging-related physical functions in *C. elegans*. Furthermore, our results indicate that autophagy genes (*bec-1*, *vps-34*, and *sqst-1*) are involved in the anti-aging effects of CBD, including neuronal aging. Our results are the first to provide the anti-aging mechanisms of CBD promoting lifespan and ameliorating neuronal aging, which forms a basis for the possible application of CBD in improving neuronal health and longevity.

## Supplementary Information

Below is the link to the electronic supplementary material.

Supplementary file1 (DOCX 701 KB)
