# Supplementary material for: Cannabidiol induces autophagy and improves neuronal health associated with SIRT1 mediated longevity
Source: GeroScience. 2022 Apr 20;44(3):1505–24. doi: 10.1007/s11357-022-00559-7 (PMC9213613; doi:10.1007/s11357-022-00559-7)

**
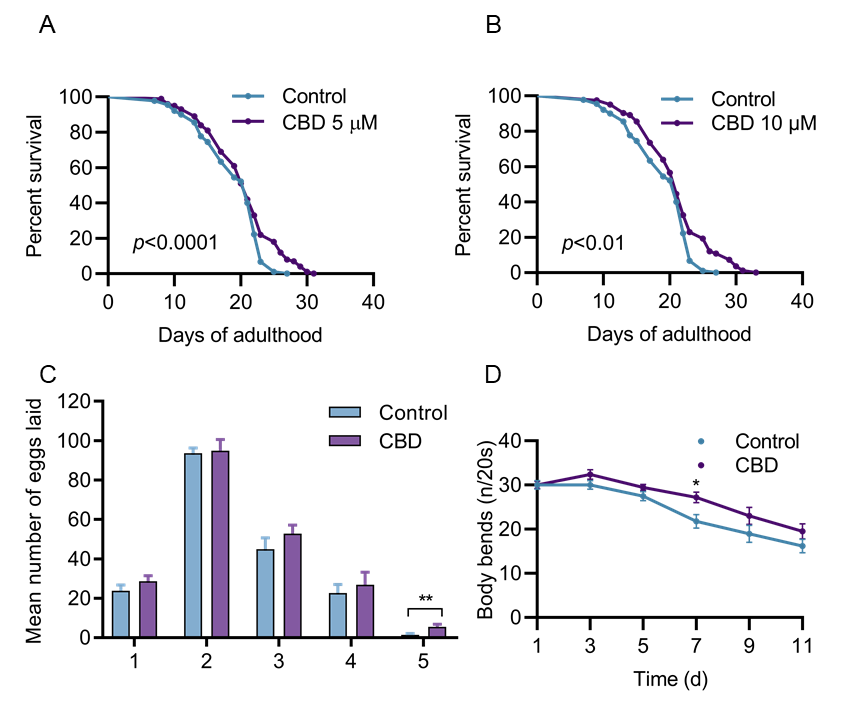
**

**Figure S1. A, B.** Lifespan of wild-type worms (N2) treated with 5 µM and 10 µM CBD, or vehicle treatment at 20℃. P values compared with control calculated using log-rank test. **C.** Comparison of eggs laid per adult animal treated with 1 µM CBD or vehicle. **D.** Rate of body bends at days 1, 3, 5, 7, 9, and 11 of adulthood in worms treated with 1 μM CBD or vehicle, n = 15-20 animals per group. Values are mean ± SEM. **P ≤* 0.05 by one-way ANOVA or two-way ANOVA followed by Bonferroni post-tests.

**
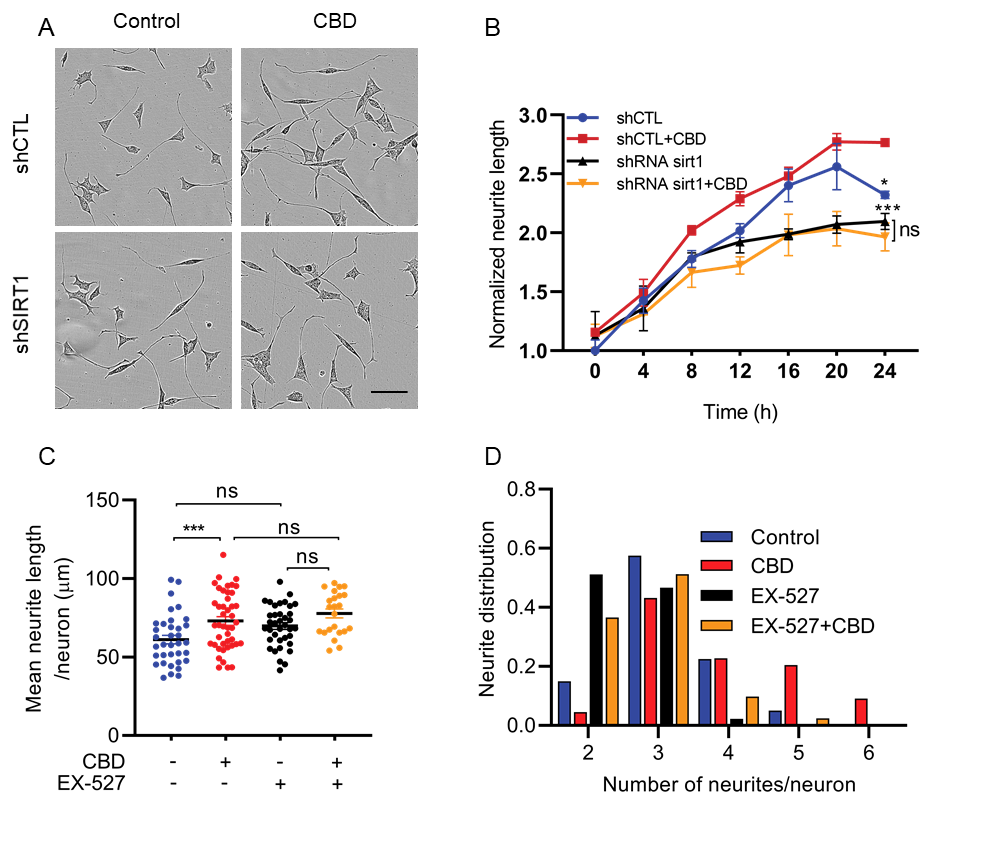
**

**Figure S2.** **A.** Representative phase-contrast images from SH-SY5Y cells observed for 24 h after transfected with shRNA control (shCTL) and shRNA SIRT1 (shSIRT1) treated with or without CBD. Scale bar = 100 μm. **B.** Neurite length in SH-SY5Y neurons was scored in four groups during 24 h. Sample sizes are shCTL, 48 cells; shCTL+CBD, 60 cells; shSIRT1, 45 cells; shSIRT1+CBD, 49 cells; 3 wells/cultures per treatment. Values are mean ± SEM. **P* ≤ 0.05, *** *P* ≤ 0.001 by One-way ANOVA.**C.** Quantification of mean neurite length in four groups (Control, 35 neurons; CBD, 44 neurons; EX-527, 37 neurons; EX-527+CBD, 23 neurons) in primary hippocampal neurons. **D.** The number of neurites per neuron was scored in four groups and calculated as a fraction of the total neurons scored for each genotype (Control, 40 neurons; CBD, 44 neurons; EX-527, 45 neurons; EX-527+CBD, 41 neurons) in primary hippocampal neurons. ns = not significant. ***P* ≤ 0.01.

**Table S1** *C. elegans* strains used in this study


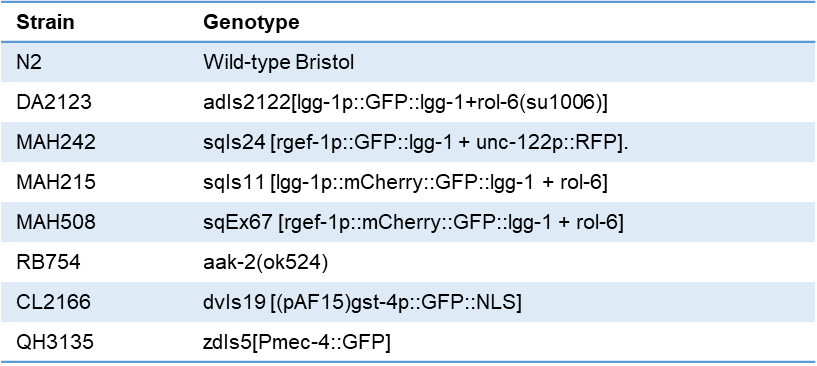


**Table S2** Summary of the *C. elegans* lifespan experiments.


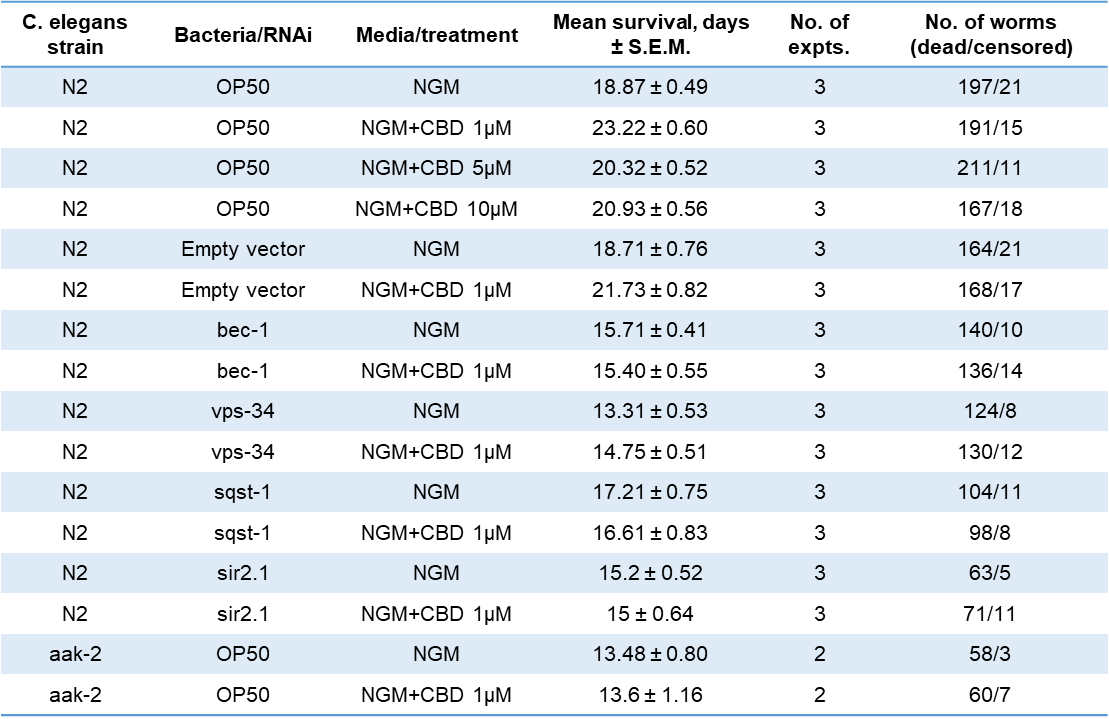


**Figure S3.** All uncropped western blots of this study.


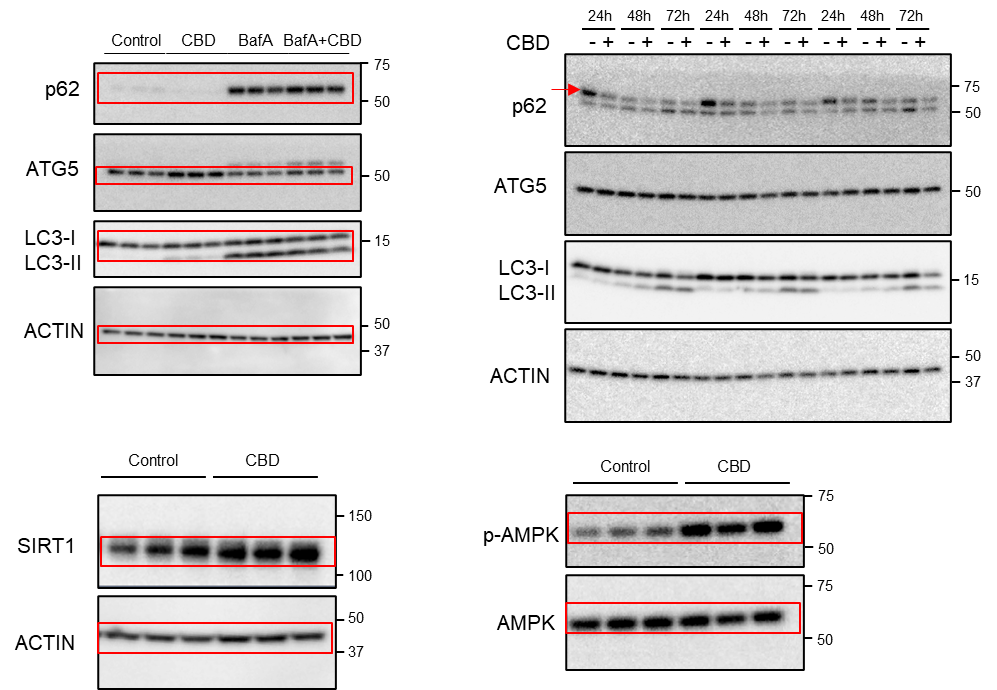

Supplement: Supplementary file 1 — Supplementary file1 (DOCX 701 KB) [file 11357_2022_559_MOESM1_ESM.docx]
